# Does the Establishment of Sustainable Use Reserves Affect Fire Management in the Humid Tropics?

**DOI:** 10.1371/journal.pone.0149292

**Published:** 2016-02-17

**Authors:** Rachel Carmenta, George Alan Blackburn, Gemma Davies, Claudio de Sassi, André Lima, Luke Parry, Wlodek Tych, Jos Barlow

**Affiliations:** 1 Forests and Governance, Center for International Forestry Research, Bogor, West Java, Indonesia; 2 Lancaster Environment Centre, Lancaster University, Lancaster, United Kingdom; 3 Forests and Livelihoods, Center for International Forestry Research, Bogor, West Java, Indonesia; 4 National Institute for Space Research, São José dos Campos, SP, Brasil; Instituto de Pesquisas Ecológicas, BRAZIL

## Abstract

Tropical forests are experiencing a growing fire problem driven by climatic change, agricultural expansion and forest degradation. Protected areas are an important feature of forest protection strategies, and sustainable use reserves (SURs) may be reducing fire prevalence since they promote sustainable livelihoods and resource management. However, the use of fire in swidden agriculture, and other forms of land management, may be undermining the effectiveness of SURs in meeting their conservation and sustainable development goals. We analyse MODIS derived hot pixels, TRMM rainfall data, Terra-Class land cover data, socio-ecological data from the Brazilian agro-census and the spatial extent of rivers and roads to evaluate whether the designation of SURs reduces fire occurrence in the Brazilian Amazon. Specifically, we ask (1) a. Is SUR location (i.e., *de facto*) or (1) b. designation (i.e. *de jure*) the driving factor affecting performance in terms of the spatial density of fires?, and (2), Does SUR creation affect fire management (i.e., the timing of fires in relation to previous rainfall)? We demonstrate that pre-protection baselines are crucial for understanding reserve performance. We show that reserve creation had no discernible impact on fire density, and that fires were less prevalent in SURs due to their characteristics of sparser human settlement and remoteness, rather than their status *de jure*. In addition, the timing of fires in relation to rainfall, indicative of local fire management and adherence to environmental law, did not improve following SUR creation. These results challenge the notion that SURs promote environmentally sensitive fire-management, and suggest that SURs in Amazonia will require special attention if they are to curtail future accidental wildfires, particularly as plans to expand the road infrastructure throughout the region are realised. Greater investment to support improved fire management by farmers living in reserves, in addition to other fire users, will be necessary to help ameliorate these threats.

## Introduction

Wildfires in tropical forests pose an important threat to biodiversity and rural livelihoods and directly impact global environmental change through increasing carbon emissions [1–4]. They have become increasingly prevalent across the humid tropics in recent decades [5], and are now 5–11 times more frequent than the estimated natural fire regime in parts of the Brazilian Amazon [6]. At least 85,500 km^2^ of forest in the southern Brazilian Amazon were estimated to have burned between 1999 and 2010 [7].

Within the Amazon forest, these forest fires are promoted by a combination of climatic events, forest degradation and human activities. Fire seasons are particularly severe when climatic changes linked to El-Niño events and the Atlantic Multidecadal Oscillation extend the dry season [8,9], while forest flammability is increased by human disturbance events such as selective logging, fragmentation and previous fires [6,10,11]. However, it is local human activities that provide the ignition sources that cause most forest fires [12], including land clearance linked to colonization and agricultural expansion and the use of swidden agriculture [13,14].

Given the significance of fire-reduction for mitigating climate change, reducing biodiversity loss, and safeguarding local livelihoods, it is vital that effective fire management strategies are developed that protect tropical forests. Protected areas are one of the leading tools used to alleviate environmental pressure on the planet's remaining natural biomes [15]. In Brazil, they serve different roles, including biodiversity conservation, safeguarding land rights and rural livelihoods, or dual objectives related to achieving economic and environmental outcomes [16]. Sustainable Use Reserves (SURs, IUCN categories V and VI) cover over 905,000 km^2^ and are of particular interest for forest fire management in Brazil. While they contain large areas of forest with high biodiversity and carbon stocks, their inhabitants are typically smalholders (including forest and river extractavists) for whom fire-use remains a crucial tool in their swidden farming systems (also known as shifting cultivation and slash-and-burn) [16]. Other forms of land management requiring fire, including pasture creation and maintenance, may also be of increasing concern in SURs [17,18].

There is considerable incertitude about the effectiveness of SURs in reducing fire use or controlling fire management, and little is known about the impact of reserves on fire dynamics (compared against a pre-designation baseline). First, none of the previous research effort examines the impact of reserve *creation* on the spatial density of fires, nor on local fire management practices. Creation impact, i.e., a *de jure* shift born by policy change, is important to assess in order to understand the effectiveness of such an intervention independent of pre-existing landscape attributes (e.g. [19]). Second, few studies incorporate the temporal relationship between fire and rainfall [20,21] and none do this specifically to evaluate reserves. Yet, from an environmental perspective, fire management (i.e. when you burn, relative to rainfall events and wind speed etc,), may be more important than aggregate measures of fire occurrence due to the higher risk of fire escape (i.e. transition from intentional agricultural fire to accidental wildfire) in drier conditions. Furthermore, there is evidence that smallholders use environmental cues such as rainfall as an active fire management practice [22–25]. Third, much of the research effort evaluating the effectiveness of protected areas focusses on deforestation (e.g. [19,26–29]) or hunting (e.g [19,26–28,30,31]), while relatively few studies address fire occurrence (e.g. [32–37]). These studies are often limited in the inference they can make on reserve effectiveness due to reliance on direct comparisons between reserve and buffer areas or reserve and “everywhere else” [38]. Finally, the studies that do examine fire activity offer mixed evidence regarding the effectiveness of SURs at ameliorating fire. Studies show that fire activity may be lower in reserves relative to the surrounding landscape [34,39], yet the inverse can also occur [36]. Other studies highlight the importance of the institutional model (such as funding & reserve type) and contextual features of the landscape such as remoteness (e.g. distance to fluvial and road networks) and human population density [26,32,33,35]. Nelson & Chomitz [33] use matching techniques to control for variables influencing fire and find SURs outperform strict protected areas. However, they do not include a pre-protection measure of fire activity.

We reduce the uncertainty surrounding the policy performance of SUR creation on fire use and management by evaluating fire-activity in 49 SURs in the Brazilian Amazon, compared to the wider landscape surrounding the reserve (a control buffer area of 10km) and apply a pre-protection baseline. We untangle the effects of policy intervention (i.e. reserve creation) from the differences in landscape attributes (e.g. land cover, distance to rivers, distance to roads, population density) in order to understand if observed differences in spatial fire density or fire management between reserves and buffers are a result of *de jure* shifts in legal status, or are in fact, *de facto*. We do this by measuring the difference in fire density before and after reserve creation, which controls for temporal variability in fire occurence, while modelling the impact of landscape attributes on fire. In addition we assess if a shift in fire management practices occurred following reserve creation. Using a pre-protection measure as a baseline is especially policy-relevant given the on-going creation of SURs in the Brazilian Amazon [40]. Our approach allows us to address the limitations associated with reserve versus buffer comparisons. Specifically we account for non-random location bias and temporal shifts in fire occurrence, which can overestimate impact [21,38].

The SURs analysed include agroextractive settlement projects (PAEs—Portuguese acronym) and extractive reserves (RESEXs—Portuguese acronym), two essentially similar models (PAEs were the original extractive reserves, created in response to lobbying from figures such as Chico Mendes, [16]), yet that are administered by entities with particularly distinct remits. PAEs are administered by the National Institute of Agrarian Reform (INCRA), whose prime aim is colonization and agrarian reform, while RESEXs are regulated by the Chico Mendes Institute of Biodiversity Conservation (ICMbio) and emphasize an environmental focus. The contested priorities, ideologies and politics of the larger institutional structures that regulate these units can influence the management effort directed at improving the sustainability of the socio-ecological system within them [17]. We assess these two SUR models separately in order to understand if differentiated constraints of the institutional models exist in order to identify where additional support may be most effectively directed. Specifically, we examine the following research questions, (1) a. Is SUR location (i.e. *de facto*) or (1) b. designation (i.e. *de jure*) the driving factor affecting performance in terms of the spatial density of fires? In particular, we evaluate how reserve type, access (distance to roads and rivers) and population density explain fire occurrence in reserves and their surrounding buffers, and whether reserve creation (the establishment of PAEs and RESEXs) affects fire density in the reserves compared to 10 km areas (buffers) surrounding the reserves. Next we ask, (2) Does reserve creation affect fire management (i.e. the timing of fires in relation to rainfall)? We draw on our analyses to assess the implications for the future management of SURs in Amazonia.

## Materials and Methods

### Data sources

#### Reserve, fire and rainfall data

**Fig 1:**
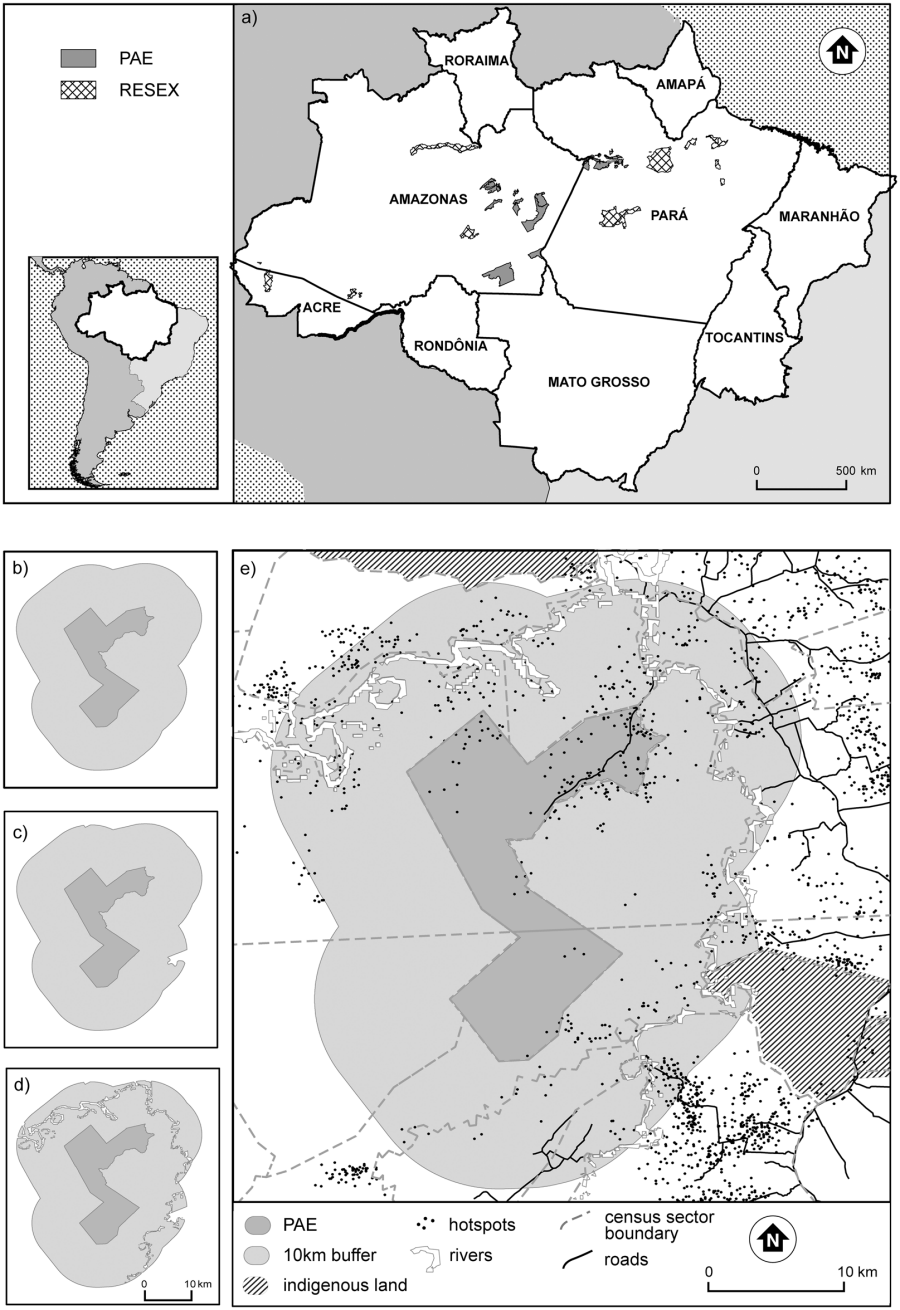
Study sites and geospatial data preparation. The Legal Brazilian Amazon (a) showing the Federal sustainable use reserves (n = 49) decreed between 2004 and 2006 that were the foci of this study. These sustainable use reserves include `Agro-Extractive Projects`(PAEs—Portuguese acronym) and `Extractive Reserves`(RESEXs). The reserve denoted by an arrow is highlighted as an example PAE with (b) 10 km buffer, (c) indigenous lands removed (d) with the area of rivers removed, and (e) showing hotspots in relation to the PAE and buffer for the study period (2001–2009) and other layers (i.e. roads and population data) used within the analysis.

The location of Federal RESEXs, PAEs and Indigenous Lands in the Legal Brazilian Amazon were acquired from the Brazilian National Institute for Space Research (INPE) (Fig 1). Land cover data was obtained via Terraclass and was supplied at 30m resolution from the Brazilian Agricultural Research Corporation (Embrapa). These data pertain to 2008, and we assume that land cover changes from 2007 to 2008 were negligible. River data were originally compiled by INPE and included all watercourses >100m wide based on dry season water levels. Population data were sourced from The Brazilian Institute of Geography and Statistics (IBGE) 2007 census. The road network data includes official and non-official roads detected in 2007 and were digitized and made available by the Amazon Institute of People and the Environment (IMAZON). Including measures of population and access allow us to separate *de jure* and *de facto* impacts on fire and we do this using both an area offset measure and spatial fire density.

Fire data used in the analysis were based on daily hotspot/active fire locations for the entire Legal Brazilian Amazon via the MODIS (or Moderate Resolution Imaging Spectroradiometer) Data Processing System (MODAPS, v5.1), sourced from the Fires Information for Resource Management (FIRMs) web interface [41]. Hotspot recognition employs the algorithm developed by Giglio et al., [42], where fire locations are recorded when one or more fires (≥227°C) are identified within a 1 km^2^ pixel. The validation exercise by Schroeder et al, [43], undertaken in the Amazon confirmed the accuracy of the MODIS fire algorithm. Their data indicate that over the range of fire types and intensities experienced in the Amazon, approximately 13% of a 1 km^2^ MODIS pixel needs to be occupied by an active fire in order to achieve a high detection probability. While smaller areas of fire can generate a hotspot, and MODIS can routinely detect fires of an average 30m x 30m, the detection probability decreases. Hence, in the context of the present research, MODIS hotspot data provided a proxy for the occurrence of biomass burning events associated with agriculture, forest conversion and the maintenance of previously-deforested areas [43].

We minimise the detection differences found to exist between satellites capable of fire detection [44] by focussing on MODIS only, which is carried on NASA’s Aqua (EOS PM) and Terra (EOS AM) satellites. These capture both afternoon (e.g. agricultural, land clearing and pasture maintenance fires) and evening burns (e.g. the land management fires and accidental fires) in the Amazon. Although hotspot data includes a mixture of forest and non-forest agricultural and accidental fires (as data were not partitioned by land cover), both can be seen to be environmentally undesirable burnings, and contained agricultural fires provide a potential wildfire ignition source. Hotspot counts underestimate fires since cloud cover, heavy smoke and forest canopy cover impedes their detection [45].

We obtained daily data on all hotspots detected between 01.01.2001–31.12.2009. Although Aqua was launched in July 2002, this does not affect our analyses since in cases where analysis was not restricted to 2007, we standardise detections within reserves by their occurrence outside (i.e. using the difference in fire density between reserves and their buffer areas). We acquired area averaged daily rainfall data for the same temporal period from the tropical rainfall measuring mission data, version 6 (TRMM 3B42-v6) at 0.25° spatial resolution from the Interactive Online Visualization ANd aNalysis Infrastructure (Giovanni) web interface using the geographic extents of reserves (RESEX and PAEs) and control buffer zones obtained from INPE. Aragão et al., [46] have shown that the TRMM data provide an accurate estimation of rainfall patterns in the Amazon region. We assumed that rainfall would be relatively consistent within reserves and their buffer zones. Thus we were able to account for driving factors related to drought [25].

#### Data processing

We created bands of 10 km surrounding each reserve (referred to as “buffer” throughout). Buffers served to validate our observations of reserve performance against a control area of fire occurrence within the wider landscape [29,47,48]. To account for additional factors affecting reserve performance and avoid an incomplete assessment of reserve performance by a simple comparison of reserves and buffer areas, we include measures of land cover, human population density and accessibility in our analysis in addition to an evaluation of reserve establishment. Land cover was calculated using the Tabulate Area tool in ArcGIS and the percentage area of forest and non-forest areas (e.g. *cerrado*) within reserves and their buffers was computed. Human population density was calculated for each census sector based upon the 2007 census data. While the uncertainties of Modifiable Areal Unit Problem (MAUP) are problematic to such measures, we follow others in adopting this approach and assume that the contribution of spatial density estimates outweigh its limitations at the spatial scale of our analysis [37,49]. As there was not perfect congruency between reserve boundaries and census sectors, mean population density values were calculated for reserves and buffers separately (Fig 1). The data were then converted into a raster surface of 500 m resolution, a gridded approach similar to Bueno et al [50]. Zonal statistics were then used to calculate the mean population density per zone (i.e. reserve or buffer). Given the scarcity of improved estimates or bottom-up data applicable for the large research area of concern, our approach assumes homogenous population density within each census sector with the caveat that this may incur overestimates of populations within reserves. Following the creation of a Euclidean distance surface, zonal statistics were also used to calculate the mean distance to roads (official and non-official) and to rivers for each reserve and each buffer (i.e. the mean of all cells within the reserve or buffer). Higher rural population densities are associated with higher fire prevalence since almost all fires in Amazonia are anthropogenic [12]. Accessibility is related to fire occurrence through various direct and indirect pathways. For example, fluvial and road networks provide access to forests for logging, facilitate colonization, agricultural expansion and urban-based farming and make commodity markets more accessible to local producers [51,52].

To avoid assessing the performance of areas with ambiguous institutional status, we removed areas where Indigenous Lands overlapped with reserve or the control buffer zones, or where these zones overlapped between reserves. River areas were excluded from reserve and buffer area calculations (Fig 1). All spatial analysis was performed in ArcGIS^®^ software by Esri.

To analyze the effect of policy intervention, i.e. reserve creation, on the timing of fires, we acquired hotspot and rainfall data pertaining to both before and after reserve creation. We included only those reserves created between 2004–2006. A total of 36 Federal PAES and 13 RESEX were retained for analysis (Table 1).

**Table 1:** Extractive reserves and agro-extractive settlement projects in the Brazilian Amazon created between 2004 and 2006.

Reserve type	Reserve name	Year	Area (km^2^)	FOR_in (%)	FOR_out (%)	HHD (km^2^)	PD (km^2^)	Roads (mean, m)	Rivers (mean, m)
**RESEX**	Arapixi	2006	1291.08	98	94	0.17	0.84	37830.60	4268.99
	Arióca Pruanã	2005	837.56	91	79	0.48	2.64	4455.42	5904.6
	Gurupá Melgaco	2006	1432.11	93	96	0.42	2.52	28099.30	6391.72
	Ipau Anilzinho	2005	533.79	48	60	0.28	1.28	3741.69	2250.88
	Lago do Campaña Grande	2004	2981.73	97	97	0.06	0.39	17600.60	15403.9
	Lago do Cedro	2006	0.60	-		0.00	0.00	224309.00	224309
	Mapuá	2005	924.54	88	94	0.33	1.86	39305.30	15403.9
	Rinzho do Liberdade	2005	3215.12	99	97	0.09	0.48	34225.30	5904.86
	Rio Iriri	2006	7348.29	-	-	0.01	0.03	23243.90	7118.75
	Rio Unini	2006	3005.40	98	98	0.01	0.05	18796.70	7185.67
	Riozinho do Anfrisio	2004	1928.20	-	-	0.45	2.52	26231.80	4364.93
	Terra Grande Pracuuba	2006	8061.65	86	91	0.02	0.12	96158.00	5989.08
	Verde Para Sempre	2004	12676.51	76	68	0.12	0.62	20610.40	5908.79
**PAE**	Abacaxis	2005	2762.85	99	98	0.16	1.15	98430.10	5200.20
	Abacaxis II	2005	16.25	97	92	0.27	1.35	57214.60	4295.18
	Acará-Acu	2006	10438.14	0	1	0.01	0.09	20634.70	2484.77
	Aripuana-Guariba	2005	85.13	98	89	1.47	7.95	18980.30	8228.98
	Aritapera	2006	123.08	0	20	0.86	4.46	13096.10	1083.28
	Atumá	2006	745.75	0	3	1.00	5.63	13469.73	1188.05
	Cabaliana I	2006	940.37	56	67	0.71	3.47	6995.70	3328.46
	Cabaliana II	2006	91.52	94	84	0.51	2.24	21032.50	5706.68
	Cacoal Grande	2006	170.48	0	11	0.00	0.00	15106.90	2158.67
	Eixo Forte	2005	481.50	52	52	0.46	2.33	514.07	5498.57
	Inajá	2005	27.06	92	77	0.00	0.00	17856.02	4993.76
	Ituqui	2006	846.15	22	48	0.10	0.61	5938.59	691.56
	Juruti Velho	2005	2663.11	73	77	0.97	5.56	2255.90	6478.58
	Lago Grande	2005	70.95	55	66	1.61	8.34	4409.08	6275.28
	Madalena	2006	2.64	0	0	1.58	7.35	16401.90	1045.64
	Maria Tereza	2006	1182.10	33	33	0.11	0.56	3580.45	769.69
	Maripiti	2006	52.32	96	87	2.69	14.65	19032.90	7126.00
	Missionario Rufino	2005	350.63	53	48	0.37	1.81	5153.51	1213.46
	Novo Jardim	2005	86.10	92	83	0.65	4.01	11704.60	914.95
	Oncas	2006	38.16	47	82	1.21	5.56	18987.80	1032.98
	Paraná de Baixo	2006	16.58	0	20	0.46	2.09	3812.85	1569.50
	Paru	2006	698.17	0	17	0.54	2.72	11908.00	492.65
	Piranha	2005	2023.65	39	60	0.02	0.12	19524.00	4503.36
	Salé	2006	95.79	4	15	0.81	3.36	7260.05	789.58
	Salvcão	2006	35.06	0	24	0.22	1.33	15011.50	1862.14
	Santa Rita	2006	40.43	0	2	0.71	3.59	9838.47	6472.05
	São Benedito	2005	167.89	95	93	1.53	9.31	3544.53	6258.47
	São Pedro	2006	40.22	0	22	0.00	0.00	17950.80	2717.96
	Tapara	2006	75.21	10	24	0.14	0.91	8589.24	1375.17
	Terra Firme	2005	68.15	95	90	0.96	4.66	9664.62	5145.96
	Tres Ilhas	2006	694.38	0	12	0.20	0.96	9578.02	715.40
	Trocana	2005	1294.36	94	88	0.01	0.05	16325.60	5577.71
	Tupuna Igopo-Acu	2005	13.73	99	97	0.00	0.00	9358.69	6061.73
	Urucuíituba	2006	166.03	0	10	0.77	3.84	12711.00	500.76
	Vale do Salgado	2005	11.27	26	41	0.55	2.89	4183.40	3832.06
	Valhá-Me deus	2006	2762.85	0	4	0.16	1.15	16786.20	917.94

Extractive reserves and agro-extractive settlement projects in the Brazilian Amazon created between 2004 and 2006, year of reserve creation (Year) is given. Reserve area (Area km^2^) refers to area after the removal of indigenous lands and rivers. Data for household density (per km^2^) (HHD) and population density (per km^2^) (PD) were obtained from the 2007 census. Distance to roads and distance to rivers are for 2007 only. Land cover data is from Terra Class (2008) and gives percentage forest cover within reserves (FOR_in) and their buffers (10km) (FOR_out). Sources: For cencus data see: http://www.ibge.gov.br/english/estatistica/economia/agropecuaria/censoagro/2006/; for land cover data see http://terraclass.cpatu.embrapa.br/; for PAEs see; http://www.incra.gov.br/sites/default/files/uploads/reforma-agraria/questao-agraria/reforma-agraria/relacao_de_projetos_de_reforma_agraria.pdf; for RESEXs’ see: http://www.icmbio.gov.br/portal/unidades-de-conservacao.html.

Each hotspot was assigned to the reserve or buffer zone within which it was detected. Hotspots overlapping a zone were associated to both (i.e. counted once for each zone) and an area offset measure using fire counts was used for research question (1)a. (i.e. is SUR performance *de facto*?) (Table 2). The total number of hotspots in each zone was divided by the total area to give spatial fire density (the number of fires. km^-2^). Values of the difference in spatial fire density were then used as our dependent measure for research question (1)b. (i.e. is SUR performance *de jure*?) (Table 2). The temporal density of fire (or likelihood of fire as defined by the probability of occurrence on any given day) was applied in research question (2) (i.e. does reserve creation affect fire management?).

**Table 2:** Research questions applied and the data and time frame pertaining to the analytical approach.

	Research question	Data	Time frame	Analysis
1.a.	Is fire density best explained by reserve location (*de facto*)?	Reserve type and buffer areas Distance to roads Distance to rivers Population density Fire counts	2007	GLMM
1.b.	Does SUR designation (*de jure*) affect reserve performance in terms of spatial fire density?	Reserve type and buffer areas Difference in fire density (between reserve and buffer)	2001–2009	GLMM
2.	Does reserve creation affect fire management (i.e. the timing of fires in relation to rainfall)?	Reserve type and buffers TRMM rainfall data Fire temporal probability density	2001–2009	Time series

An overview of the research questions addressed in the present study combined with information on the data used in each analysis. Time frames and analytical approach are also given. Full sources for all data can be found in Table 1 and data processing and preparation is treated in the text.

### Data analysis

Analyzing the performance of protected areas against their immediate buffer zones, or the surrounding landscape at large, has been challenged due to the importance of often overlooked landscape attributes (such as connectivity, population density) in conditioning reserve performance [38]. To overcome these hurdles and account for important confounders in our analyses, we first check the balance of our sample, and then use these results to inform the selection and inclusion of covariates to consider in our analyses.

We observe that most variables (HHD, PD, river) have similar means, suggesting that these factors are already well balanaced in our sample (Table 3.). However, the lack of significant differences may be in part because of the small sample size (49 reserves). The standardized or normalized differences (computed as (mean_treated—mean_control)/sqrt((var_treated + var_control)/2) and compared to the recommended 10% criterion [53,54]), are widely preferred measures of balance because they are less directly influenced by sample size [55], and are nearly all greater than the 0.1 cut-off suggested by Austin [53]. This balance assessment suggests that the distributions are different, challenging the balance suggested by the similar means. It is notable that for two potentially importante confounders—forest cover and distance to roads, we observe a good balance between reserves and buffers. To aid account for differences between inside and outside, we retain these measures in all our analyses, and only exclude them from the final models if they do not improve model fit.

**Table 3:** Summary of covariate balance between reserves inside and buffers.

	N	p>|t|	Norm Diff	Var ratio (Int/Con)	KS P-value (BS)
Forest cover (%)	49	0.593	0.080	1.321	0.104
HHD (km^2^)	49	0.320	0.202	1.188	0.364
PD (km^2^)	49	0.421	0.163	1.402	0.262
River (mean, m)	49	0.348	-0.191	140.99	0.156
Road (mean, m)	49	0.721	-0.072	1.012	0.366

Covariate balance for all variables included in the analysis between inside reserves and their buffer areas (10km). Data for forest cover (%), household density (per km^2^) (HHD), population density (per km^2^) (PD) and the mean distance (m) to rivers (River) and roads (Road). T-test on means, normalized differences ((we compute them as (mean_treated—mean_control)/(sqrt((var_treated + var_control)/2)), variance ratio and bootstrapped (n = 500) Kolmogorov-Smirnov test are presented. P-values are not significant (at p<0.05), indicating equality of means. Normalized differences great than 0.1 indicate unequal distribution of the data between control and intervention.

Given the importance of the existing proportion of forest and non-forest cover (e.g. *cerrado*) is likely to contribute to the fire dynamics in the landscape [37], we further examine the differences in land cover between reserves and buffer zones. We already observed similar averages between forest cover inside and outside the reserves. Additionally, forest and non-forest cover were highly correlated both inside and outside the reserves (t = 13.43, p<0.0001, R^2^ > 0.8). However, given the potential important influence of variations in land cover on fire dynamics, we initially included the proportion of forest cover and non-forest cover (sequentially) in all models, and subsequently removed them for model simplification when not significant.

#### Temporal and “snap-shot” analyses

To address the first research question—concerning the extent to which fire density is explained by *de facto* characteristics, or *de jure* reserve status we analysed reserve performance based on fire data for the year 2007 while including landscape attributes (Table 2). This model specified the effectiveness of reserve, reserve type, human population density, distance metrics (mean distance to proximate rivers and roads) and forest cover, on the occurrence of hot-pixels (fire counts) detected in reserves and their buffers in 2007. 2007 was selected for the snap-shot analysis as census and road data were collected in this year. In addition, 2007 was an extreme drought year in the Brazilian Amazon allowing for an interpretation of reserve performance in high stress years [8]. Because we used fire counts we fitted the model using Generalized Linear Mixed Models with a Poisson error term using the glmer function in the ‘lme4’ package for R [56], and applied the “offset” function to control for reserve size (i.e. to account for the potential sampling bias). We included reserve identity as a random factor to account for spatial autocorrelation, as well as an observation-level crossed random effect to account for the presence of over-dispersion in the original model. The final model was obtained through stepwise model simplification until no further decrease in AIC scores could be obtained.

The second model analysed the temporal fire data (2001–2009) to assess the impact of reserve creation on the difference in spatial fire density between a reserve and its buffer zone (i.e. to assess evidence of a shift before and after reserve creation) (Table 2). Using the difference in spatial fire density provided a relative measure of reserve performance which also controls for temporal variation in fire occurrence (e.g. borne by climatic factors) and accounts for differences in weather conditions between geographic areas [21]. This was necessary because, for example, fire densities might be increasing in all areas, but to a lesser extent in reserves relative to buffers. The model was fitted with difference in spatial fire density (i.e. between a reserve and its buffer area) predicted by reserve type, reserve creation, forest types and their interaction as fixed factors. Forest cover was removed from the final model as it was not significant and decreased the model fit. Given the longitudinal nature of the data for each reserve, and the non-equal before and after reserve creation years, we used the nlme package for Linear Mixed-Effects Models [57] including reserve identity as random effect and gaussian error, since data were normally distributed. All analyses were performed in R 3.1.2.

#### Time series: fire management, rainfall and institutional factors

Time series analysis was used to examine the impact of reserve creation on fire management (research question (2)) (Table 2). We used model residual variance as a proxy for fire management, inferring that higher residuals in our model of fires and API were indicative of factors other than rainfall (and dryness) influencing fire management decisions, using the following procedures.

*Time varying probability density*: The time varying probability density (a formal measure of time varying likelihood of the fire occurrence on any day, further referred to as likelihood) of the fires occurrence was estimated using Maximum Likelihood (ML) optimised cubic splines, which are commonly used to describe time-varying densities of discrete processes. The data used here were the daily MODIS hotspot values. This specific implementation of the data-based estimator uses an Integrated Random Walk model and its ML optimisation included in the Captain Toolbox for Matlab [58]. Conditional densities were calculated, that is the number of fires in each time-step divided by the total number of fires in that zone. We denote the estimated temporal probability density of fires as: *p*(*t*), where *p(t)* is the likelihood (or temporal probability density) of fire occurrence in period *(t*,*t+*Δ*t)* where Δ*t* is one day and *t* is time measured in days. The notation *p*_*k*_ is used to simplify *p(k*Δ*t)* denoting *k*-th time sample/step—the day number in the series. This notation will be used throughout the paper.

*Isolating rainfall influence*: We assumed that MODIS-detected fires were not accidental and that their occurrences are affected by the decisions of reserve inhabitants [12,22–24]. Decisions regarding when to burn are driven by rainfall events and “other factors”. Since we cannot reliably separate these “other factors” we assumed that they are related to the reserve status due to associated factors such as secure land tenure, impacts on community cohesion, increased inputs from extension agencies and increased awareness of rules and sanctioning. Our model splits the likelihood of fires occurring on any given day into the ‘natural’, wetness driven component, and the ‘other factors’ driven one. We obtain the overall likelihood obtained from MODIS data as described in (*i*) above with the best fit of the wetness driven likelihood estimate.

We modelled the wetness, rainfall driven component of the fire likelihood as a function of the Antecedent Precipitation Index (API). API is a dynamic index commonly used in hydrology [59] to describe the wetness of the catchment based on the knowledge of previously occurring rainfall (here—derived from TRMM). We used API for the same reason as applications in hydrology—it adequately describes the catchment wetness with very few assumptions. API has one parameter (ignoring the scale—irrelevant in this case)—interpreted as the memory, time constant, or recession parameter, reflecting temperature and evaporation. As the parameter is estimated from the data, no assumptions are required. Typical responses of API to a rainfall event are given in supplementary information (S1 Appendix).

Fires occur almost exclusively in periods where the API is below a specific threshold level due to fuel flammability, and are more likely when the catchment is drier (API is lower). We therefore proposed the following model for the ‘natural’ component of the likelihood of fires, which takes into account the observed relationship with the API and produces as output a dynamic estimate of the fires temporal probability density *p(API)* attributed to the rainfall effects.

$$p(API_{k})=\{\begin{array}{cc} 0\;when: & API_{k}\geq \theta \\ a\cdot (\theta -API_{k})\;when: & API_{k}<\theta \end{array}$$

where *a* is a proportionality constant and *θ* is the reaction threshold, meaning that for *API_k_* > *θ* (catchment wetter than the threshold value) there are no fires. Notation for parameter optimization are given in supplementary information (S1 Appendix).

The hypothesis that reserve status influences fire management practices in favour of less risky burns (i.e. fires occur in moister catchment conditions than previously) was supported where reserve creation was linked to a change in the model fit, i.e. when the rain explained a significantly different proportion of the fire observations following reserve creation (comparing *before* and *after*). Changes in the proportion of natural (API driven) vs “other factors” driven components of likelihood are measured as proportion of the variance contributions to the time varying likelihood. A “worse fitting” API driven model after the legislation change compared to before the change can be interpreted as higher effectiveness of the reserve. This was done for all reserves and buffers to provide an indication of the effectiveness of reserve creation. All the time series analysis was conducted in Matlab 7.12 using Captain Toolbox 7.4.

## Results

### Spatial analysis of reserve effectiveness to prevent fire in 2007

**Fig 2:**
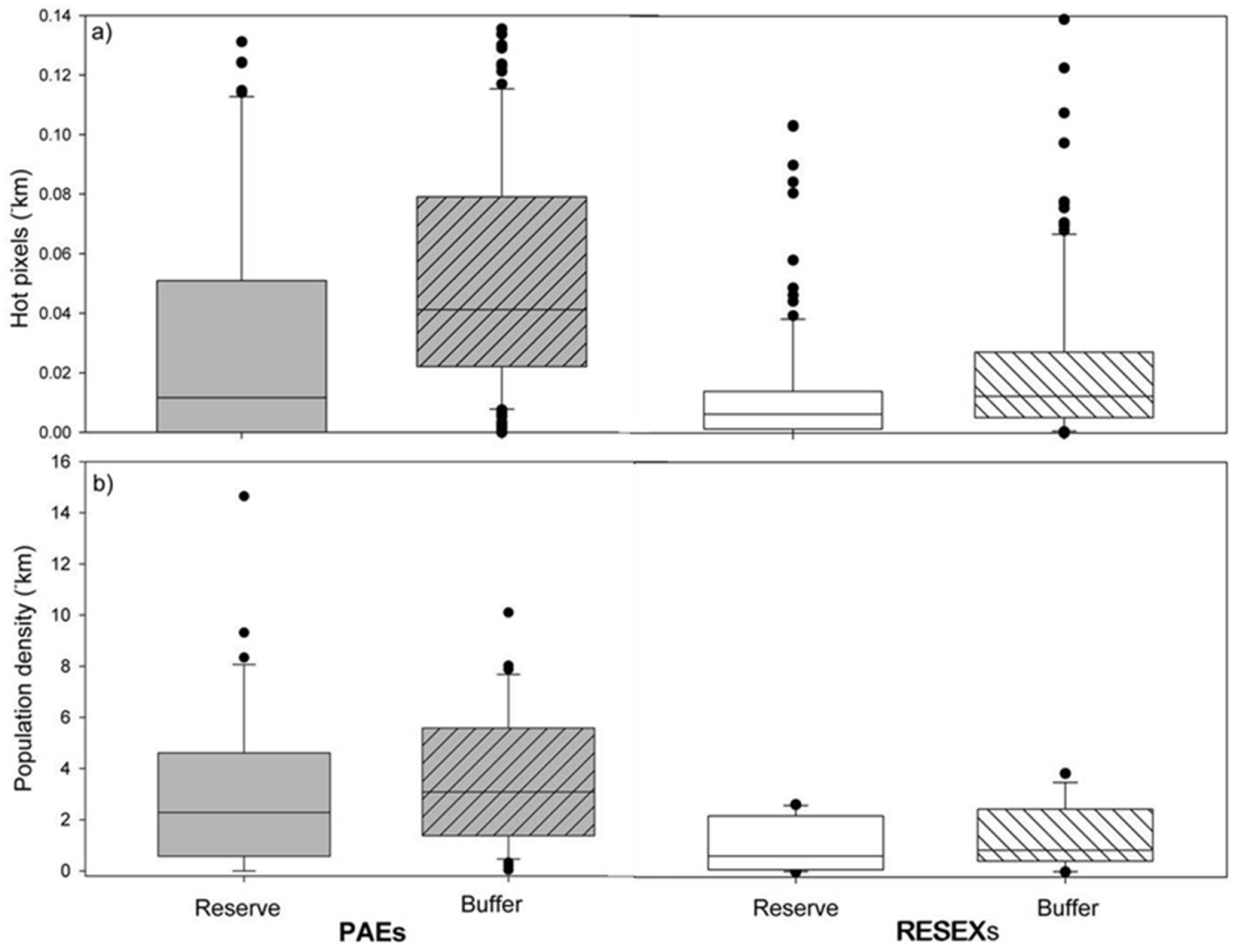
Spatial fire and human population density. Fire density and human population density in study sustainable use reserves. Fire density (a) and human population density (b) in Federal sustainable use reserves (SURs) (PAEs, n = 36; RESEXs, n = 13) and their buffer zones (10 km radius) in the Brazilian Amazon. Whiskers indicate the 10^th^ and 90^th^ percentiles, outliers are indicated with black dots. Fire density is MODIS hotspots km^-2^ between 2001–2009. Human population density is (people km^-2^ in 2007) sourced from the IBGE 2007 census.

A total of 3,021 hot pixels were recorded by MODIS during 2007 within the reserves and 10 km wide buffer. Spatial fire density was, on average, highest in PAE buffers (mean fires. km^-2^ ± SD = 0.05 ±0.08), and PAE reserves (0.04 ±0.09) and lower in RESEX buffers (0.02 ± 0.02) and RESEXs (0.01 ± = 0.01) (Fig 2a). The regression model indicated a significantly higher incidence of fires outside the reserve (i.e. in buffers), and fires were positively correlated with population density and proximity to rivers, reflecting a positive relationship to connectivity in the landscape (Fig 2b). Interestingly, road distance was not significant, likely reflecting a high degree of collinearity with population density which provided a better model fit. Finally, we found a weak significance of reserve type predicting the fire count, with the RESEXs having lower fire occurrence (Table 4).

**Table 4:** Fire occurrence in Federal sustainable use reserves in Legal Brazilian Amazon created between 2004–2006 and their buffer zones (10 km radius).

	Estimate	Std.Error	z-value	Pr(>|z|)	
(Intercept)	-4.91	0.30	-16.11	<0.001	***
Reserve Type	-0.73	0.40	-1.81	0.070	.
Inside-Outside	0.82	0.21	3.98	<.001	***
Pop. Density	0.11	0.06	2.06	0.040	*
River	0.04	0.01	7.15	<.001	***

Model results for fire occurrence (hot-pixel counts) in Federal sustainable use reserves in Legal Brazilian Amazon created between 2004–2006 and their buffer zones (10 km radius). Fire counts were from 2007 and regressed against: reserve type; zone (reserve or buffer (IO) respectively; population density (km^-^2); average distance to river (km) (River). Interaction term between reserve type and zone, distance to roads and forest cover were initially included in the full model and dropped during model simplification.

### Temporal analysis from 2001–2009: the impact of reserve creation

We used longitudinal hot-pixel data from 2001 to 2009 to assess the impact of reserve designation and reserve type on spatial fire density. A total of 28,343 hot pixels were recorded over this period in reserve and buffer zones and available in the MODAPs database. However, we found that neither reserve creation (t = 0.97, p = 0.333) nor reserve type (t = -1.14, p = 0.262) had a significant influence on the difference in spatial fire density.

### Fire management, rainfall and reserve creation

**Fig 3:**
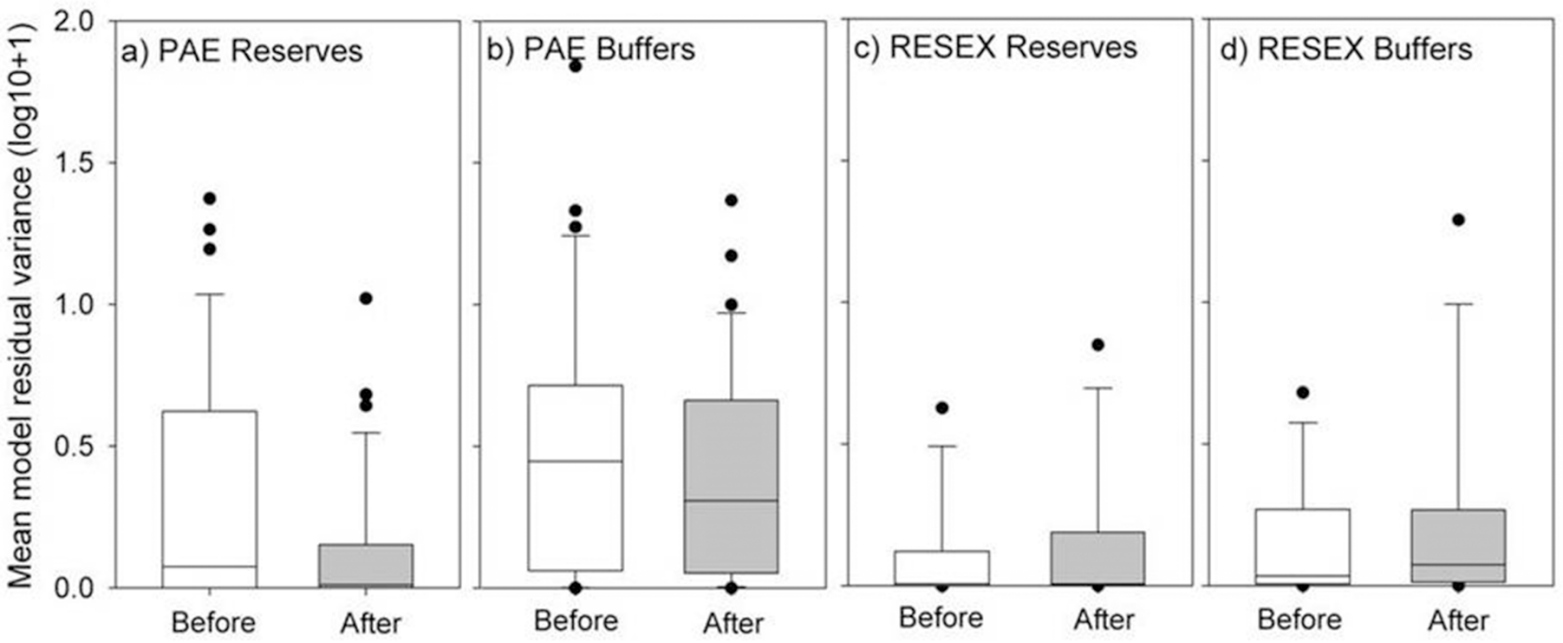
Amazonian smallholder fire management. Amazonian smallholder fire management (timing of fires in relation to rainfall) in sustainable use reserves. Model residual variance output (log10+1) for the models of antecedent precipitation index (API) and fire likelihood before and after creation of Federal sustainable use reserves (SURs) in the Brazilian Amazon, including (a) PAEs and (c) RESEXs and their buffer zones (10 km radius, panels b and d respectively). Reserve creation was only significant in the PAE buffer zones.

We tested for an impact of reserve creation and SUR type on fire management (i.e. the timing of fires in relation to rainfall) within reserves and buffers. We used model residual variance as a proxy for fire management, inferring that higher residuals in our model of fires and API were indicative of factors other than rainfall (and dryness) influencing fire management decisions. Reserve creation did not induce any shift towards improved fire management by SUR residents (i.e. in the form of less risky burnings) (Fig 3a and 3c). Reserve creation showed a marginal but negative impact in PAEs, where fires had a slightly stronger association with dry periods following reserve establishment (Fig 3a). No discernible impact on the timing of fires was identified for RESEXs (Fig 3c) or related buffer zones (Fig 3b and 3d respectively).

### Synthesis of spatial and temporal analysis to assess reserve performance

**Fig 4:**
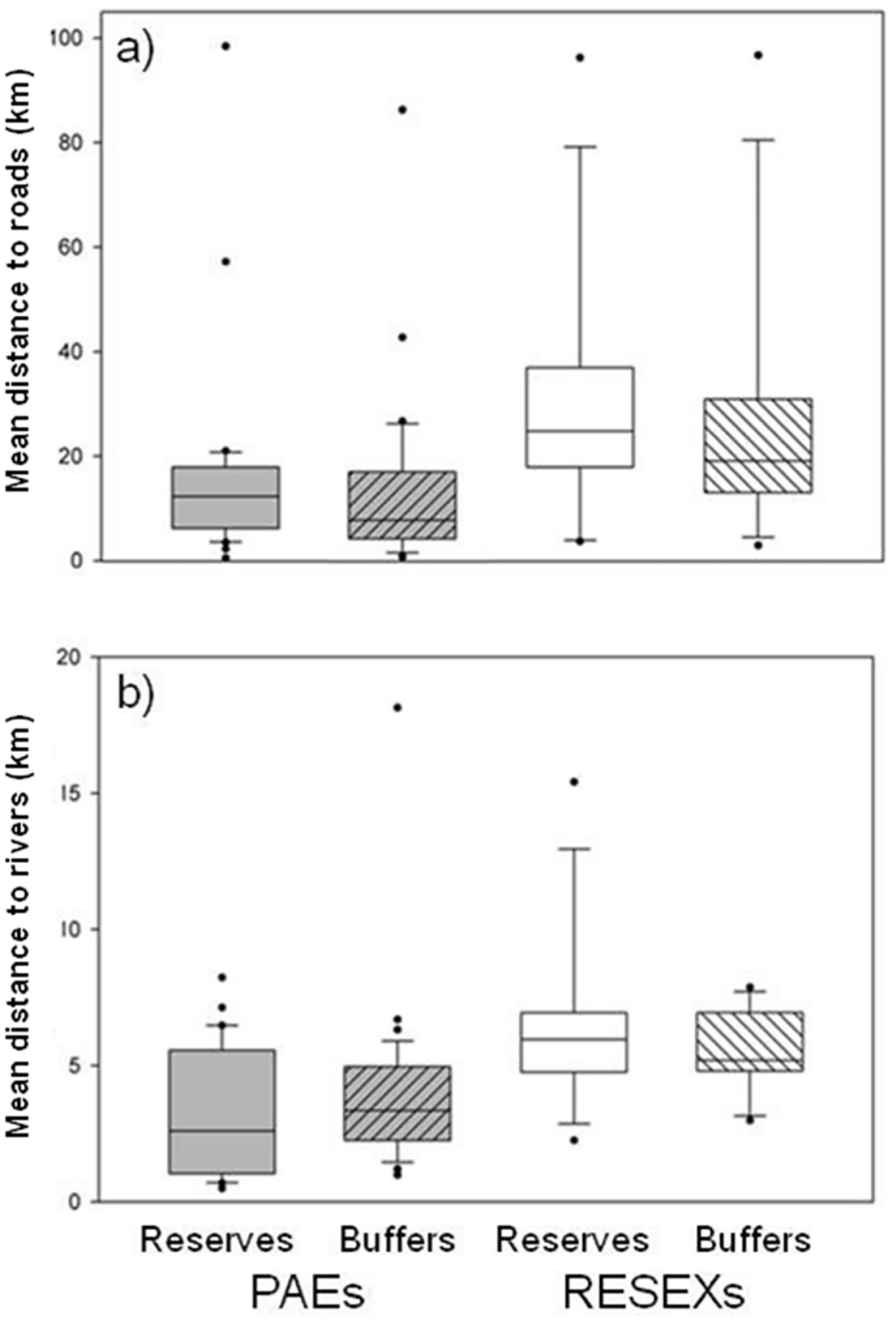
Accessibility of sustainable use reserves. Sustainable use reserve accessibility in the Brazilian Amazon. Distance of Federal sustainable use reserves (PAEs, grey boxes, n = 36; RESEXs, clear boxes, n = 13) (no dash) and their buffer zones (10 km radius) (dashed) to (a) rivers (all water courses over 100 m width) and (b) roads (i.e. the mean distance in km of all grid cells within the zone). River data were sourced from INPE, road data were sourced from IMAZON.

Although hotspot data indicated that SURs experienced a lower spatial density of fires than the surrounding landscape (Fig 2a), our analysis using both spatial density measures and an area offset measure, suggest that these differences were not related to reserve creation or institutional design (i.e. RESEXs or PAE) (Table 1), but instead to the very different landscape contexts (settlement and accessibility) in which the SURs were created (Table 4). On average, PAEs and their buffer zones had somewhat higher population densities (mean, [SD] in PAEs = 3.15 people. km^-2^, [3.27]; PAE buffers = 3.64 people. km^-2^, [2.62]) than the RESEX counterparts (mean, [SD] in RESEX = 1.68 people. km^-2^, [2.85]; RESEX buffers = 3.21 people. km^-2^, [7.17]) (Fig 2b). In addition, on average PAEs were more accessible by road and river than RESEXs (Fig 4a and 4b.). Overall both types of reserves are more integrated to the river network than the road network.

## Discussion

Our results show that sustainable use reserves (SURs) protect Amazonian forests from wildfires largely because of their *de facto* characteristics, as previously shown for deforestation [44]. That is, they were established in regions that tend to be remote and sparsely inhabited. Reserve creation itself, i.e. a formal *de jure* change of status, had no impact on spatial fire density. In addition, there was no evidence of improved fire management following reserve creation. Our analysis builds on evidence suggesting that less contextualized assessments of reserves and buffers are inadequate for assessing reserve performance [28,33]. In addition to separating the *de jure* (status) and *de facto* (location) factors, we demonstrate the importance of assessing the policy intervention against a pre-protection benchmark, in order to understand reserve performance. We discuss our results in light of protected area management, payments for environmental services, plans for infrastructure development in the Amazon and potential future research directions.

### Reserve performance reflects favourable local context

Fires are prevalent in the densely populated buffer areas, which tend to be more integrated with river and road networks. This is important, as studies of reserve performance (e.g. [34,48]) which do not account for differences in contextual factors, neither explicitly test for an impact of reserve creation, may link reserve performance to policy impact when in fact they are identifying *de facto* differences that exist between reserves and their buffers. Further, we show that PAEs are under higher pressure from fire since they are in more populated and accessible areas than RESEXs.

### SURs and fire management

Sustainable use reserves have a mandate towards sustainability (with different degrees of social, environmental and economic focus) and are anticipated to improve forest and livelihood outcomes. Theoretically, confirming property rights and access to land should reduce fire management risk [60]. It is therefore unexpected that we found no evidence of SUR creation leading to a shift towards lower-risk fire management (i.e. burning in moister conditions), and some evidence to the contrary in the case of PAEs. It may be that this is explained by PAE creation being accompanied by a re-configuration of ownership that can leave residents feeling ambiguous about their tenure. Such perceptions were expressed by smallholders in two SURs during focus group and participatory mapping exercises [61]. This might result in smallholders optimizing their land clearing (by burning in the drier periods) to claim use and ownership before institutional changes take place. The observed performance failure of reserve creation may be related to a lack of outreach, capacity building and support to residents following the *de jure* creation, or to the incongruence of external rules with local management capacities and burning needs [22,24]. It may also relate to lag effects, with it taking longer than 5 years for institutional differences to manifest themselves in changes in management. We emphasize the need to ground-truth these remotely sensed relationships between rainfall and fire-use. Upon validation, these approaches could provide a way of assessing fire-management across large spatial scales, supporting the implementation of payments for ecosystem services linked to good fire management (c.f. [62]).

### Implications for the future environmental performance of SURs

The potential contribution of SURs to forest conservation and climate change mitigation [63] could be being compromised by the fire-use and fire management decisions of their inhabitants. With anticipated climate change, prolonged droughts and road expansion [52,64], the sustainable use reserve network currently appears underequipped to mediate this threat to the forests and livelihoods which they aim to protect. Relatively small-scale attempts to promote fire-free agriculture in Amazonia (e.g. Inga Alley Cropping [65] and *Roça sem Queima*, [66] have found some success, yet the substitution of fire technology remains unlikely for a large segment of small-scale farmers who are unable to access such initiatives, credit and alternative technology [67] and is highly unlikely to be implemented across more remote areas of Amazonia. As large-scale transitions from fire-based swidden to fire-free agriculture are not imminently anticipated [67], improving smallholder fire management is a preferable short-term management option.

While fire frequency is a measure of human impact, fire management behaviour (i.e. the propensity to adopt measures to contain a burn) is important and may be determined by household and community characteristics [68] as well as technical capacity and environmental conditions [20,22]. We suggest there is some potential to utilize funds generated from payment for ecosystem service (PES) initiatives such as REDD+ to provide incentives and opportunities for smallholders to improve fire management [62]. Indeed some projects related to reduced emissions from deforestation and degradation (REDD+) are attempting to include improved fire management (e.g. [69].

Due to the difficulties of obtaining appropriate data across time periods, we do not test for changes in human population density inside of, or outside of, the reserves which represents a caveat to this work. The centrality of human settlement to fire activity suggests that there is a need to better understand the spatial distribution of population density within reserves and their trajectories (including population growth and net-migration flows), and how reserve establishment may affect this variable [70,71]. In general, more remote areas have low and decreasing human settlement compared with accessible areas in Amazonia [72]. However, it is not clear whether dynamics in SURs follow similar trends and more research is needed [50]. Further, PES within reserves may act as pull factors, increasing population density and therefore influencing fire activity within reserves.

Our results highlight the importance of connectivity in determining fire activity. SURs in this analysis are currently more connected to the river network, but plans to expand mining and pave more of the Amazonian road network [52] could place additional pressures on SURs that are currently relatively inaccessible [50,73]. Environmental Impact Assessments relating to road expansion within SURs will need to clearly identify effective measures of fire mitigation (both in terms of avoiding fire use and improving fire management) if forest integrity, species conservation and human wellbeing are to be maintained in these units. In our sample of SURs, PAEs will likely be faced with more challenges than RESEXs due to their location (Fig 4), and will therefore require extra policy attention to encourage behavioural shifts. Potentially improved road access associated with PAEs could be capitalized on to benefit fire-reduction, as it could facilitate the provisioning of machinery to reduce agricultural fire dependence.

Although our results provide evidence to suggest that reserve establishment is not affecting fire-use, the study took place at broad spatial scales and was restricted to within five years of reserve creation. It may be that reserve creation is effective over longer-time periods, or that the coarse-scale of our analysis obscures many examples of positive change. Moreover, many other scale related socio-institutional, topographic and climatic factors that were not measured here, are likely to determine fire use and land management behaviour of smallholders within reserve units (e.g. availability of equipment, funds, investments in the land) [25,35,68,74]. It is therefore important to acknowledge these limitations and refrain from assigning solutions that are not evidence-based [75]. Instead, we believe that our results should be interpreted as a stimulus for more research to develop effective policies that incentivise good fire management in sustainable use reserves and other areas of the Amazon where swidden agriculture dominates. In particular, governments and NGOs should focus on improving levels of support to smallholders, particularly in extreme drought years [22].

## Conclusions

Sustainable use reserves are an important tool for conserving tropical biodiversity and carbon stock while supporting rural populations. However, the designation of sustainable use reserves in the Brazilian Amazon has not yet provided an adequate measure to reduce fire activity in the region, or generate behavioural shifts in fire management by smallholders. We illustrate the importance of assessing policy intervention against a baseline, pre-intervention measure where this is available. Rather than being related to reserve establishment, fire activity seems to be determined by *de facto* human settlement patterns and accessibility to transport networks. Importantly, our results suggest that favourable assessments of reserve performance could be related to these *de facto* attributes, i.e. of reserves being created in locations with lower opportunity costs [27], and reserve creation has no impact. We argue that sustainable use reserves will better achieve their goals of conservation and sustainable development if greater efforts are made to improve the fire management of smallholders and if the fire implications of future road creation and population increases are adequately managed.

## Supporting Information

S1 Appendix Antecedent precipitation index typical response to a rainfal event and parameter optimization. (DOCX)
