# Supplementary material for: Does the Establishment of Sustainable Use Reserves Affect Fire Management in the Humid Tropics?
Source: PLoS One. 2016 Feb 17;11(2):e0149292. doi: 10.1371/journal.pone.0149292 (PMC4757533; doi:10.1371/journal.pone.0149292)
Supplement: S1 Appendix — (DOCX) [file pone.0149292.s001.docx]

**S1 Appendix. Antecedent precipitation index typical response to a rainfall event and parameter optimization.**

A typical response of the Antecedent Precipitation Index (API) to a series of rainfall events *R_k_* ( *k=1,…,*n where *n* is the number of time steps - days in this case - in the data set) has the following form:

$$API_{k+1}=\alpha\cdot API_{k}+(1-\alpha)\cdot R_{k}$$

where alpha is the memory parameter, typically between 0.4 (for fast recession) and 0.95 (slow recession) and a full range between 0 and 1, and k is the day number as in the consideration of the time varying fire probability density. The interpretation of this model is that of a first order difference equation: for alpha of 0.85 means that 85% of the catchment wetness on day k persists till day k+1 if there is no rain on day k. This relationship generates an exponential decay curve of API in response to a single rainfall event with the time (decay) constant of:

$\tau=\frac{-1}{\ln(\alpha)}$ days

Parameters $\left(\alpha,a,\theta\right)$ are optimised for each data set (rainfall, fire density) to generate the best fit between observed *p_k_* and modelled *p(API_k_)*, by minimising:

$$Q(\alpha,a,\theta)=\sum_{k=1}^{N} (p_{k}-p(API_{k}))^{2}$$

Optimisation if performed for each location data set, as it reflects the parameters of the locality such as land cover, vegetation type and density, weather conditions etc, all affecting evaporation
